# Sample representativeness and influence of attrition on longitudinal data collected as part of a national medical career tracking project

**DOI:** 10.1186/s12909-023-04472-1

**Published:** 2023-07-25

**Authors:** Charlotte J. W. Connell, Alexander J Salkeld, Cameron Wells, Antonia C. Verstappen, Phillippa Poole, Tim J Wilkinson, Warwick Bagg

**Affiliations:** 1 School of Medicine, Faculty of Medical and Health Sciences, The University of Auckland, Auckland, New Zealand; 2 Otago Medical School, University of Otago, Christchurch, New Zealand; 3 The Centre for Medical and Health Sciences Education, Faculty of Medical and Health Sciences, The University of Auckland, Auckland, New Zealand

**Keywords:** Medical education, Medical schools, Longitudinal studies, New Zealand, Workforce, Curriculum

## Abstract

**Background:**

The Medical Schools Outcomes Database and Longitudinal Tracking Project (MSOD) in New Zealand is one example of a national survey-based resource of medical student experiences and career outcomes. Longitudinal studies of medical students are valuable for evaluating the outcomes of medical programs against workforce objectives. As a prospective longitudinal multiple-cohort study, survey response rates at each collection point of MSOD vary. This paper assesses the effects of participant non-response rates on MSOD data.

**Methods:**

Demographic variables of MSOD respondents between 2012 and 2018 were compared to the distribution of the demographic variables in the population of all NZ medical graduates to ascertain whether respondent samples at multiple survey collection points were representative of the population. Analysis using logistic regression assessed the impact of participant non-response on variables at collection points throughout MSOD.

**Results:**

2874 out of a total population of 2939 domestic medical students graduating between 2012 and 2018 responded to MSOD surveys. Entry and exit surveys achieved response rates around 80% and were broadly representative of the total population on demographic variables. Post-graduation survey response rates were around 50% of the total population of graduates and underrepresented graduates from the University of Auckland. Between the entry and exit and the exit and postgraduation year three samples, there was a significant impact of non-response on ascribed variables, including age at graduation, university, gender and ethnic identity. Between the exit and postgraduation year one sample, non-response significantly impacted ascribed and non-ascribed variables, including future practice intentions.

**Conclusion:**

Samples collected from MSOD at entry and exit are representative, and findings from cross-sectional studies using these datasets are likely generalisable to the wider population of NZ medical graduates. Samples collected one and three years post-graduation are less representative. Researchers should be aware of this bias when utilizing these data. When using MSOD data in a longitudinal manner, e.g. comparing the change in career intentions from one collection point to the next, researchers should appropriately control for bias due to non-response between collection points. This study highlights the value of longitudinal career-tracking studies for answering questions relevant to medical education and workforce development.

## Introduction

New Zealand (NZ) has two medical schools, one at the University of Auckland and the other at the University of Otago. From 2012, all NZ medical students have been invited to participate in a longitudinal study known as the Medical Schools Outcomes Database and Longitudinal Tracking (MSOD) project. As with any longitudinal design, there is variability in response rates at each collection point, which may impact on how the results can be interpreted. The impact of such variability is the focus of this paper.

Previous workforce surveys have typically examined cross-sections of medical workforce demographics and distribution, but these do not provide sufficient insight into the mechanisms resulting in these characteristics of the medical workforces examined, nor the effectiveness of interventions aimed at addressing workforce imbalances [1]. The aim of the MSOD project is to investigate the influence of individual characteristics, curricula and training on eventual career patterns in the medical workforce in New Zealand. This is described in more detail in Poole et al. [1]. Students are invited to complete surveys at several time points. The first survey invitation is at entry to medical school (Commencing Medical Students Questionnaire; CMSQ), then at completion of medical school (Exit Questionnaire; EQ). The remaining invitations are follow-up questionnaires one, three, five and eight years after graduation (PGY1; PGY3; PGY5; PGY8).

To date, the MSOD project holds over 16,000 survey responses describing demographics, intentions for medical discipline and location of future practice, and factors influencing career decision-making at the various stages of the participants’ medical training. The MSOD project is ongoing and informs the development of curricula, educational interventions and policy.

The MSOD project has high response rates at the early survey invitation time points (CMSQ and EQ), which, coupled with the large class sizes (approximately 300 commencing students at each medical school in 2021), results in a large and potentially highly representative sample. At these time points, it is common to achieve greater than 80% response rates recommended for epidemiological research [2–4], which are typically regarded as the benchmark rates needed to provide the best chance of obtaining a representative sample. However, this is neither necessary nor sufficient evidence that a sample is representative of the population from which it is drawn. Representativeness of the sample is crucial for researchers to have confidence in the validity or accuracy of the findings and to be able to generalise the study results to the population from which the sample was drawn. However, if the response rates are lower than 80%, the longitudinal design and non-random sampling method that the MSOD project employs makes it susceptible to potential problems associated with declining participation [5]. Note that this is not necessarily the same as participant attrition – participants who do not respond to a particular survey may still respond to a later survey and are not excluded from the dataset. Doctors are making and acting on decisions about where and what work will be undertaken, thus declining participation is particularly pertinent at the early survey collection points after graduating from medical school (PGY1 and PGY3). As participants progress into their careers, response rates to the MSOD surveys at the postgraduate collection points drop below 80%. This may result in particular groups of people being lost in subsequent data collection, and introducing the risk of a biased sample as a result of non-response [2].

Another complicating factor is that non-response in later survey rounds in longitudinal studies can be driven by changes in the personal circumstances of the participants. This may further bias the key study variables, as these are expected to be driven in some way by these changes and the data lost to non-response in these cases would have contained this important information [6], further incentivising high response rates. Non-response rates greater than 20% may present a threat to the validity of a longitudinal study’s findings, yet there is evidence to suggest that although incomplete data might influence population estimates, estimates of association are rarely affected, thereby not resulting in misleading findings [7]. Thus, it is critical to go beyond simply reporting survey response rates and undertake systematic analysis – first to determine the degree of attrition between timepoints in longitudinal studies and then to assess the impact of the increasing non-response rate by making a comparison between responders and non-responders on key study variables [2, 8]. The findings from such an analysis allow for study results utilizing these variables to be appropriately judged in terms of their generalisability [2]. Therefore, the aim of this study is to systematically assess the effects of participant non-response rates on longitudinal data collected as part of the MSOD project. As the MSOD project is currently one of very few studies in the niche of longitudinal tracking of career intentions, this verification of sample representativeness is useful not only to highlight areas that need to be addressed by end-users of this dataset but to also inform the interpretation of these data from similar current and future longitudinal career tracking studies. The exploration of the presence of non-response bias is an important, and sometimes overlooked, step of analysis for users of such data sets [9, 10].

As a limitation, the subsequent implementation of typical methods for handling missing data due to non-response, including weighting adjustments for questionnaire non-response and imputation for question or unit non-response [11] are beyond the scope of this study.

## Methods

In this study, the demographic variables of MSOD respondent groups (outlined below) were compared to the distribution of the demographic variables in the finite population of all NZ medical graduates who graduated between 2012 and 2018, in order to ascertain whether the MSOD respondent groups at different collection points were broadly representative of the population. Following this, an analysis was conducted to assess the impact of participant drop-out/attrition on study variables at various collection points throughout the MSOD project (up to PGY3). In each case, inference is taken with respect to a quasirandomization model, where it is assumed that initial non-response, and subsequent attrition, occur as the result of random processes.

### Study sample and respondent groupings

The survey responses collected from medical students when they begin study at medical school (CMSQ), at graduation (EQ), and one (PGY1) and three (PGY3) years after graduation were extracted from the MSOD project database for the study sample. The sample for this study comprised domestic University of Auckland and University of Otago medical students who graduated between 2012 and 2018, inclusive (n = 2874). International students were excluded from the study sample.

The study sample was organized into the following respondent groups: ‘CMSQ responders’, ‘EQ responders’, ‘PGY1 responders’, and ‘PGY3 responders’. Each of these groups comprised the participants who responded to the survey at each respective data collection point. These groups substantially overlap one another, as a large number of participants have responded to multiple questionnaires.

### Data collection, groupings and variables

To ascertain whether the respondent groups were broadly representative of the overall population, the demographics of the respondent groups were compared to the demographics in the population of all NZ medical graduates (2012–2018). Following this, an analysis was conducted to assess the impact of participant attrition on study variables at various collection points throughout the MSOD project (up to PGY3).

For the initial representativeness analysis, the reference population (comparator group) for all timepoints was every NZ medical student who graduated from 2012 to 2018, inclusive. The study respondent groups are all necessarily a subset of this reference group. The population distribution of key demographic variables for this group was determined by combining publicly available data sources (‘Population distribution: NZ Graduates (2012–2018)’, Table 1). The sources were the Medical Deans Australia and NZ reports (2010–2019) [12–19], the Medical Deans Australia and NZ Student Statistics Database [20], the University of Auckland Graduate Database [21], and the University of Otago Graduate Database [22]. The demographic variables collected from these sources included: university attended; graduation year; indigeneity (Māori / non-Māori), and gender. The project database derived this same information for the MSOD respondent groups.

**Table 1:** **Distribution of the characteristics of domestic NZ medical graduates (2012–2018) versus the distributions of characteristics of NZ Medical School Outcomes Database and tracking project (MSOD) respondents**. The population reference group is *Population distribution: NZ graduates (2012–2018)*. Chi-square goodness-of-fit tests compared the distribution of characteristics in the MSOD responders groups with the population reference group. Data represent graduate counts (percentage of total). *Statistically significant based on p < α, as set by criterion power analysis. α < 0.00002. ^†^Category not included in chi-square comparisons. ^€^Population percentage only includes graduates who had the opportunity to respond to the PGY3 survey at time of the study (i.e. Graduates from 2012–2016, inclusive)

		Population distribution: NZ Graduates (2012–2018)	CMSQ responders (93% of pop.)		EQ responders (78% of pop.)		PGY1 responders (52% of pop.)		PGY3 responders (54% of pop.)€	
				*Sig.*		*Sig.*		*Sig.*		*Sig.*
University				*0.459*		*0.009*		***<*** α*		***<*** α*
	Auckland	1289 (44%)	1227 (45%)		1072 (47%)		437 (28%)		312 (29%)	
	Otago	1650 (56%)	1519 (55%)		1231 (53%)		1104 (72%)		766 (71%)	
Graduation year				*0.960*		*0.005*		*0.158*		*0.476*
	2012	334 (11%)	305 (11%)		254 (11%)		201 (13%)		188 (17%)	
	2013	383 (13%)	370 (13%)		332 (14%)		186 (12%)		213 (20%)	
	2014	396 (13%)	360 (13%)		286 (12%)		201 (13%)		191 (18%)	
	2015	417 (14%)	395 (14%)		354 (15%)		236 (15%)		236 (22%)	
	2016	469 (16%)	426 (16%)		399 (17%)		222 (14%)		250 (23%)	
	2017	457 (16%)	437 (16%)		305 (13%)		233 (15%)			
	2018	484 (16%)	453 (16%)		373 (16%)		262 (17%)			
Indigenous				*0.787*		*0.697*		*0.076*		*0.277*
	Māori	342 (12%)	315 (11%)		262 (11%)		157 (10%)		114 (11%)	
	Non-Māori	2597 (88%)	2431 (89%)		2041 (89%)		1384 (90%)		964 (89%)	
Gender				*0.281*		*0.204*		*0.0002*		*0.015*
	Female	1600 (54%)	1523 (55%)		1284 (56%)		911 (59%)		627 (58%)	
	Male	1338 (46%)	1222 (45%)		1018 (44%)		629 (41%)		451 (42%)	
	Gender diverse^†^	-	1		1		1		0	

The MSOD questionnaires collect more in-depth information on demographics, social characteristics, career intentions and influences, and workforce outcomes than what is available in the public reports of medical student demographics. A selection of these variables was used as explanatory variables in the attrition analyses. Ascribed variables (involuntary variables, such as those relating to the demographics of the respondent) selected for the analysis included age at graduation and population size of hometown (background). Non-ascribed variables (voluntary variables, such as those relating to career choices) selected for the analysis included relationship status and number of children/dependants, specialty preference and population size of future practice location.

### Statistical analysis

#### Assessing the representativeness of MSOD respondent groups

Chi-square goodness-of-fit tests were conducted to determine whether the participants who responded to MSOD questionnaires at different time points had the same distribution of each variable as the distribution in the reference population of all NZ medical graduates between 2012 and 2018. The null hypothesis for this test was that the distributions were similar to the reference population.

Given the large sample size, it is appropriate to adjust the α (alpha) value used to indicate statistical significance of the chi-square goodness-of-fit tests using a criterion power analysis [23]. With the total sample size set to the smallest respondent group (PGY3, n = 1130), effect size set to 0.2 (small-moderate), power to 0.95 and degrees of freedom to 5, the required alpha value was 0.00002. Statistical significance was set at α < 0.00002 for all chi-square goodness-of-fit tests, recognizing that the majority of this statistical analysis will be overpowered and will detect differences between the reference and sample distributions that are smaller than the nominated, ‘practically relevant’ effect size of 0.2.

#### Attrition analysis

A separate analysis was performed to assess the impact of the declining participation at later time points on the MSOD study. The impacts of participant attrition on three separate time intervals within the MSOD study were examined. The chosen intervals were: CMSQ to EQ, EQ to PGY1, and EQ to PGY3. Each time interval was first assessed for whether non-random sampling effects were present at the end time point, i.e., whether attrition was associated with a set of known variables or if it occurred due to random processes.

For the CMSQ to EQ interval, a logistic regression was used to model the probability of a respondent being present in the EQ sample after responding to the CMSQ to assess the presence of non-random sampling effects at EQ. For the EQ to PGY1 and EQ to PGY3 intervals, logistic regressions were used to model the probability of responding to PGY1 or PGY3 questionnaires after having responded to the EQ to assess the presence of non-random sampling effects at PGY1 and PGY3.

In the case that the regression analyses above suggested that attrition resulted in non-random sampling effects (indicated by the presence of a statistically significant regression co-efficient), the impact of the non-random sampling effects at that time point was investigated further by performing post-hoc comparisons between responder and non-responder groups using chi-square tests for homogeneity. For the procedures in the attrition analysis, statistical significance was set at α < 0.05.

### Ethics approval

For the MSOD project surveys, participants provided written informed consent. The MSOD project has approvals from the University of Auckland Human Participants Ethics Committee (#022388; #018456) and the University of Otago Ethics Committee (#07-155). These ethics approvals permit linking with medical school administrative data.

### Availability of data and materials

The raw datasets supporting the conclusions of this article are available upon request from MSOD (MSOD.project@otago.ac.nz).

## Results

The study sample included 2874 MSOD responders in total. Demographic information for responder groups at each collection time point is provided in Table 1.

### Population representativeness of MSOD responder groups

The ‘CMSQ responders’ and ‘EQ responders’ groups represented 93% and 78% of the total population of NZ domestic medical graduates (2012–2018), respectively. Chi-square goodness-of-fit tests revealed no significant differences between the population and the respondent samples (Table 1).

The ‘PGY1 responders’ group from the MSOD sample represented 52% of the population of domestic NZ medical graduates (2012–2018). Chi-square goodness-of-fit tests revealed an underrepresentation of survey responders from the University of Auckland (p < α, Table 1). There was no difference between the reference population and the PGY1 sample in the proportions of indigenous graduates, year of graduation, or gender (Table 1).

The ‘PGY3 responders’ group represented 54% of the eligible population of NZ medical graduates who could have completed the PGY3 survey at the time of the study. Chi-square goodness-of-fit tests revealed an underrepresentation of survey responders from the University of Auckland (p < α, Table 1). There was no difference between the reference population and the PGY3 sample in the proportion of indigenous graduates, year of graduation, or gender (Table 1).

### Assessing attrition effects across collection points

#### Impact of attrition between CMSQ and EQ samples

Table 2 shows the results of the logistic regression analysis to assess the presence of non-random sampling effects in the EQ sample as a result of attrition of MSOD respondents from CMSQ to EQ. The dependent variable in the analysis was whether the respondent responded to the CMSQ and EQ (EQ responders), or only responded to the CMSQ (EQ non-responders). The independent variables included in the analysis were: specialty preference at CMSQ (General Practice (GP) / Surgery / Other specialty), background (Urban / Regional / Rural / Unknown), intended future practice location as selected at CMSQ (Urban / Regional / Rural), age at graduation (25 years and under / over 25), participation in a rural program (No / Yes), university (Otago / Auckland), female gender (No / Yes), and indigenous ethnic identity (No / Yes). Relationship status and number of dependants were left out of the model due to a high number of missing cases (n = 854). The only variables observed to have significant non-random sampling effects were demographic variables (age at graduation, University, female gender, and indigenous ethnicity). Respondents who were aged 25 years or younger at graduation, from the University of Auckland, female, or non-indigenous were more likely to respond at EQ. Conversely, respondents did not appear to drop out at the EQ collection point on the basis of specialty preference or future practice location intentions.

**Table 2:** **Logistic regression: Responders versus Non-responders at MSOD Exit Questionnaire (EQ)**. Responder at EQ was the reference category for the outcome variable. Participants in the study sample who responded to the Commencing Medical Students Questionnaire (CMSQ) were input into the model. N = 2746. *p < 0.05

		No response at EQ	
Variables		Odds ratio [95% CI]	Sig.
Background			
	*Urban*	-	0.449
	*Regional*	1.2 [0.9–1.6]	0.222
	*Rural*	0.9 [0.7–1.2]	0.504
	*Unknown*	0.9 [0.4–2.2]	0.830
Future practice intention at entry			
	*Urban*	**-**	0.134
	*Regional*	0.8 [0.6–1.0]	0.091
	*Rural*	1.1 [0.8–1.6]	0.476
	*Unknown*	0.8 [0.5–1.2]	0.255
Specialty preference			
	*Other*	-	0.746
	*Surgery*	1.1 [0.8–1.4]	0.546
	*GP*	0.9 [0.7–1.3]	0.705
Age group at graduation			
	*Under 25 years*	-	
	*Over 25 years*	1.4 [1.2–1.8]	< 0.001*
Rural programme participation			
	*Not involved*	-	
	*Involved*	0.8 [0.6–1.1]	0.191
Institute			
	*University of Otago*	-	
	*University of Auckland*	0.5 [0.4–0.7]	< 0.001*
Gender			
	*Male*	-	
	*Female*	0.8 [0.6–0.9]	0.007*
Ethnic identity			
	*Non-indigenous*	-	
	*Indigenous*	1.3 [1.0–1.8]	0.043*
Constant		0.3	< 0.001*
Model Chi-square		70.7	< 0.001*
Adjusted R-square		0.04	

Table 3 shows a comparison between the EQ responders and EQ non-responders. Significant differences in proportions were found for age, university and gender. Compared to the EQ non-responders, in the EQ responders group there was a significantly lower proportion of graduates who were over 25, a higher proportion from the University of Auckland, and a higher proportion of women. The EQ responders and non-responders groups did not differ significantly in the proportions of indigenous in each group.

**Table 3:** **Comparison between Responders and Non-responders to the MSOD Exit Questionnaire**. Chi-square tests for homogeneity were performed to compare proportions between responder and non-responder groups. Each superscript letter denotes a subset of the variable categories whose within-group column proportions differ significantly as assessed by post-hoc two-proportions z-tests. N = 2746. *p < 0.05

		Within-group proportions		Sig.
		EQ responders	EQ non-responders	
Background				0.217
	*Urban*	0.67	0.66	
	*Regional*	0.12	0.15	
	*Rural*	0.2	0.19	
	*Unknown*	0.02	0.01	
	*Total*	1	1	
Future practice				0.075
	*Urban*	0.6	0.64	
	*Regional*	0.18	0.16	
	*Rural*	0.13	0.14	
	*Unknown*	0.09	0.06	
	*Total*	1	1	
Specialty preference				0.064
	*Other*	0.76	0.71	
	*Surgery*	0.15	0.19	
	*GP*	0.09	0.1	
	*Total*	1	1	
Age group at graduation				0.001*
	*Under 25 years*	0.25^a^	0.32^b^	
	*Over 25 years*	0.75 ^a^	0.68 ^b^	
Rural program participation				0.080
	*Not involved*	0.89	0.92	
	*Involved*	0.11	0.08	
Institution				< 0.001*
	*University of Otago*	0.52^a^	0.67^b^	
	*University of Auckland*	0.48^a^	0.33^b^	
Gender				0.012*
	*Male*	0.43^a^	0.49^b^	
	*Female*	0.57^a^	0.51^b^	
Ethnic identity				0.119
	*Non-indigenous*	0.89	0.87	
	*Indigenous*	0.11	0.13	

#### Impact of attrition between EQ and: PGY1 and PGY3 samples

Table 4 shows the results of the logistic regression analysis assessing the presence of non-random sampling effects in the PGY1 and PGY3 samples. The dependent variables were whether participants responded to the EQ and PGY1, the EQ and PGY3 (PGY1 responders and PGY3 responders, respectively), or only responded to the EQ (PGY1 non-responders and PGY3 non-responders, respectively). The independent variables were: specialty preference at EQ (General Practice / Surgery / Other specialty); intended future practice location as selected at EQ (Urban / Regional / Rural / Unknown); age at graduation (25 years and under / over 25); rural program (No / Yes); university (Otago / Auckland); female gender (No / Yes); dependants (No / Yes); relationship (No / Yes) and indigenous ethnicity (No / Yes).

**Table 4:** **Logistic regressions modelling the probability of no response to the MSOD surveys at Postgraduate Year One (PGY1) and Postgraduate Year Three (PGY3)**. Responder at PGY1 or Responder at PGY3 was the reference category for the outcome variables in each regression. Participants in the study sample who responded to the Exit Questionnaire (EQ) were input into the model. N = 2292 (11 observations were excluded from model due to missing relationship status). *p < 0.05

		No response at PGY1		No response at PGY3	
**Variables**		Odds ratio [95% CI]	Sig.	Odds ratio [95% CI]	Sig.
Future practice intention at Exit					
	*Urban*	-	< 0.001*	-	0.626
	*Regional*	0.7 [0.6–0.9]	0.003*	0.9 [0.7–1.1]	0.272
	*Rural*	0.5 [0.4–0.8]	0.003*	0.9 [0.6–1.3]	0.670
	*Unknown*	2.2 [1.3–3.6]	0.002*	0.8 [0.5–1.3]	0.371
Age group at graduation					
	*Under 25 years*	-		-	
	*Over 25 years*	0.9 [0.7–1.1]	0.444	1.1 [0.9–1.4]	0.189
Children or dependents					
	*No children or dependents*	-		-	
	*Has children or dependents*	1.1 [0.7–1.7]	0.647	1.1 [0.7–1.7]	0.584
Rural program participation					
	*Not involved*	-		-	
	*Involved*	0.8 [0.6–1.1]	0.263	0.6 [0.4–0.8]	< 0.001*
Institute					
	*University of Otago*	-		-	
	*University of Auckland*	5.5 [4.6–6.7]	< 0.001*	3.5 [2.9–4.2]	< 0.001*
Gender					
	*Male*	-		-	
	*Female*	0.8 [0.6–0.9]	0.005*	0.9 [0.7–1.1]	0.228
Ethnic identity					
	*Non-indigenous*	-		-	
	*Indigenous*	1.3 [1.0–1.8]	0.050	1.4 [1.1–1.9]	0.013*
Relationship status					
	*Single*	-		-	
	*Relationship*	1.0 [0.8–1.2]	0.922	0.9 [0.8–1.1]	0.288
Specialty preference					
	*Other*	-	0.309	-	0.077
	*Surgery*	0.8 [0.7–1.1]	0.146	1.2 [1.0–1.5]	0.114
	*GP*	0.9 [0.7–1.2]	0.433	0.9 [0.7–1.1]	0.218
Constant		0.4	< 0.001*	0.9	0.550
Model Chi-square		436.98	< 0.001*	237.6	< 0.001*
Adjusted R-square		0.23		0.13	

In the PGY1 regression, MSOD participants who had a regional or rural future practice intention, those graduated from the University of Otago, or participants who were female were significantly more likely to respond to the PGY1 survey. Those with an unknown future practice intention at EQ were significantly less likely to respond to the PGY1 survey. Respondents did not drop out at the PGY1 collection point on the basis of specialty preference, age at graduation, participation in a rural program at medical school, dependants, relationship status, or ethnicity (Table 4).

In the PGY3 regression, MSOD participants who participated in a rural program, those graduated from the University of Otago, or participants who had a non-indigenous ethnic identity were more likely to respond at PGY3. Respondents did not drop out at the PGY3 collection point on the basis of non-ascribed variables, such as specialty preference and future practice location, or the remaining demographic variables, female gender, dependants, relationship status and age at graduation (Table 4).

Table 5 shows a comparison of within-group proportions among the PGY1 and PGY3 responder and non-responders groups. Significant differences in proportions were found between PGY1 responders and PGY1 non-responders on future practice intentions, university and gender. The PGY1 non-responders group had a significantly higher proportion of participants with urban or unknown future practice location intentions, a higher proportion of University of Auckland graduates, and a lower proportion of graduates with a GP specialty preference compared to the PGY1 responders group. In the comparison between PGY3 responders and PGY3 non-responders, significant differences were observed in future practice intentions, rural program participation, University, gender, specialty preference and ethnicity. The PGY3 non-responders group had significantly higher proportions of participants with an urban future practice intention; graduates from the University of Auckland; and those with an indigenous ethnicity compared to the PGY3 responders group. Conversely, there were significantly lower proportions of participants in the PGY3 non-responders group with a regional or rural future practice intention; those who participated in a rural program; females; and those with a GP specialty preference compared to the PGY3 responders group.

**Table 5:** **Comparison between Responders and Non-responders to the MSOD Postgraduate Year One (PGY1) and Postgraduate Year Three (PGY3)**. Chi-square tests for homogeneity were performed to compare proportions between groups. Each superscript letter denotes a subset of the variable categories whose within-group column proportions differ significantly as assessed by post-hoc two-proportions z-tests. N = 2746. † Relationship N = 2292 due to 11 missing observations. *p < 0.05

		PGY1 within-group proportions			PGY3 within-group proportions		
Variables		Responders N = 1354	Non-responders N = 949	Sig.	Responders N = 944	Non-responders N = 1359	Sig.
Future practice				< 0.001*			< 0.001*
	*Urban*	0.56^a^	0.66^b^		0.55^a^	0.64^b^	
	*Regional*	0.32^a^	0.24^b^		0.32^a^	0.26^b^	
	*Rural*	0.10^a^	0.05^b^		0.10^a^	0.06^b^	
	*Unknown*	0.02^a^	0.05^b^		0.04	0.03	
Age group at graduation				0.271			0.283
	*Under 25 years*	0.73	0.75		0.75	0.73	
	*Over 25 years*	0.27	0.25		0.25	0.27	
Children or dependents				0.501			0.215
	*No children or dependents*	0.95	0.95		0.96	0.95	
	*Has children or dependents*	0.05	0.05		0.04	0.05	
Rural program participation				0.264			< 0.001*
	*Not involved*	0.88	0.9		0.86 ^a^	0.91^b^	
	*Involved*	0.12	0.1		0.14^a^	0.09^b^	
Institution				< 0.001*			< 0.001*
	*University of Otago*	0.70^a^	0.29^b^		0.71^a^	0.41^b^	
	*University of Auckland*	0.30^a^	0.71^b^		0.29^a^	0.59^b^	
Gender				< 0.001*			0.011*
	*Male*	0.41^a^	0.49^b^		0.41^a^	0.46^b^	
	*Female*	0.59^a^	0.51^b^		0.59^a^	0.54^b^	
Ethnic identity				0.109			0.029*
	*Non-indigenous*	0.90	0.87		0.90^a^	0.87^b^	
	*Indigenous*	0.1	0.13		0.10^a^	0.13^b^	
Relationship status^†^				0.380			0.229
	*Single*	0.49	0.51		0.48^a^	0.50^b^	
	*Relationship*	0.51	0.49		0.52^a^	0.50^b^	
Specialty				< 0.001*			< 0.001*
	*Other*	0.57^a^	0.64^b^		0.58	0.6	
	*Surgery*	0.19	0.18		0.16^a^	0.20^b^	
	*GP*	0.25^a^	0.18^b^		0.26^a^	0.19^b^	

## Discussion

This study assessed sample representativeness and influence of participant attrition on longitudinal data collected at multiple timepoints as part of a national medical career tracking project: the MSOD project. The MSOD samples collected at the start of medical school and at graduation (CMSQ and EQ) were broadly representative of the graduate population over the time period. As the response rates at these time points met the generally accepted threshold response rate of 80%, this finding is in agreement with previous literature [24–26]. Thus, findings that arise from using these datasets in a cross-sectional manner can be confidently generalised to the population of NZ graduates. The datasets collected postgraduation were representative for demographic variables, but overrepresented University of Otago graduates and underrepresented University of Auckland graduates. Researchers should be aware of this when using them in a cross-sectional manner. The analysis that assessed the impact of attrition on the datasets across time suggests that, from the start of medical school to graduation (CMSQ and EQ), respondents dropped out of the study based on ascribed variables, such as age at graduation, gender or ethnicity. From graduation to one year post-graduation (EQ to PGY1), and graduation to three years post-graduation (EQ to PGY3), respondents dropped out based on a mixture of ascribed (e.g. gender) and non-ascribed variables (e.g. career intentions at graduation). Thus, if the datasets are to be utilized in a longitudinal manner, such as comparing the change in career intentions or influences from one collection point to the next, for example, then researchers should employ analysis and modelling techniques that allow for them to control for those ascribed variables.

Furthermore, a non-responders analysis should be conducted if the samples collected in the postgraduate years are utilised as a part of the research, whether in a cross-sectional or longitudinal manner. If, as in this case, a non-response bias is discovered in the subset of data of interest, methods can be employed to adjust the dataset for this non-response. Typical methods include weighting adjustments to deal with initial non-response and attrition at a whole-questionnaire level, and imputation to deal with non-response to individual questions. These techniques are well-documented in the literature [11, 27].

In comparing the various MSOD samples to the population of NZ medical students, those collected at CMSQ and EQ were representative of the population. At the CMSQ collection point, the MSOD project sampled 93% of the graduate population collection point, and 78% at EQ collection point (Table 1). Though the proportions of graduates from each University did not differ significantly from the population in the CMSQ and EQ samples, the attrition analysis found that from CMSQ to EQ that University of Otago graduates were two times more likely than University of Auckland graduates not to respond at the EQ collection point after responding to the CMSQ (Table 2). Survey collection methods have differed between medical schools at these time points, and may explain the higher propensity for dropout in Otago graduates from entry to graduation. The University of Auckland methods at both these timepoints have historically involved inviting participants to fill out paper surveys at mass gatherings of each cohort which took place annually. Students who were not present at those and therefore not invited to complete a paper survey in person were mailed a paper survey with a pre-paid envelope to return, though the number of these students in each cohort were very small. Conversely, the University of Otago has used a digital method of survey collection at these timepoints—students received an email inviting them to participate, and a link to a digital version of the MSOD questionnaire at each collection point. Given the high response rates at each timepoint, researchers using the CMSQ or EQ datasets in a cross-sectional manner can be confident in generalising their findings to the population. If the CMSQ and EQ datasets are to be used in a paired, longitudinal manner where changes across time are of interest, then the analysis should be adjusted for the university that participants attended, along with adjusting for other ascribed variables that were significant in the attrition analysis, such as age at graduation, indigeneity and gender. It should also be noted that the different survey collection methods may introduce measurement errors into the dataset [6]. As much as possible, the questions in the MSOD surveys have been designed to have a limited range of answers that can be given and presented in a format to minimise this issue. The online questionnaires completely guide respondents through these questions, whereas paper questionnaires are open to respondents accidentally straying from the question instructions.

Statistically significant differences in the population and MSOD samples were observed for samples collected at one year and three years postgraduation. In both cases, the samples were significantly different in terms of the proportions of respondents in the sample from each university (Table 1). Although the total proportion of the population sampled at the PGY1 and PGY3 collection points was appreciably less than 80% (52% and 37% of the population for PGY1 and PGY3, respectively), the samples did not differ on any of the other variables that were compared to the population. Nevertheless, the significant difference in representation of graduates from each university in each sample compared to the population does mean that findings using these datasets are likely less generalisable to the wider population of NZ medical graduates when using these datasets in a cross-sectional manner. In both the PGY1 and PGY3 samples, there is a larger representation of students from the University of Otago, and an underrepresentation of University of Auckland participants compared to the population. It is likely that the lower representation of University of Auckland graduates in the PGY1 and PGY3 samples is due the differences in survey collection method between the Universities. As mentioned above, Auckland students at CMSQ and EQ were invited to complete the survey in person at a mass cohort gathering at entry or graduation, whereas at the PGY1 and PGY3 collections the first cohorts were initially invited to respond via a mail-out paper survey for a few years of data collection, before switching to distributing a link to a digital version of the surveys. The University of Otago have kept their collection method the same at all of the collection points for the EQ, PGY1 and PGY3 surveys, while only the CMSQ survey collection has switched from a mass cohort gathering to digital distribution of links to the survey. Although response rates to CMSQ and EQ are lower for University of Otago (92% and 75%, respectively) than the University of Auckland (95% and 83%, respectively), response rates to the PGY1 and PGY3 collection time points remain high from University of Otago graduates (67% and 46%, respectively), whereas there is a sharp drop in response rates from University of Auckland graduates (34% and 24% for PGY1 and PGY3, respectively). It is a recognised effect that changing from initial in-person collection events to self-administered online collection at later collection points can contribute to survey nonresponse [6] – in this case potentially stemming from the unfamiliarity of the participants with the online survey questionnaires and collection process. Additionally, the change in survey collection method from a mail-out paper survey to online distribution may be expected to introduce measurement errors.

The attrition analysis on the postgraduate samples revealed a drop-out of respondents from EQ to PGY1 or PGY3 on a mixture of both ascribed variables, such as indigeneity or gender, and non-ascribed variables, such as participation in a rural program at medical school, or future career location intentions. Of note in the attrition analysis for the PGY1 sample was the presence of drop-out based on the preferred location of future practice that participants selected at EQ. The PGY1 responders had significantly lower proportions of those who selected a preference for Urban practice, or did not respond to that question, and higher proportions of those who selected a preference for regional or rural practice than the PGY1 non-responders (Table 5). The practice location intentions of graduates are of great interest to researchers who have utilised the MSOD project data [28–30], and those interested in how those intentions change between medical school and postgraduate practise one year out of medical school should be aware that the sample collected at this timepoint is biased towards graduates with a stated intention at the end of medical school for Rural or Regional practice. This bias could have carry-over effects into other career intentions and preferences, such as specialty interests and the influence of various factors in career decision-making. In the PGY3 sample, graduates who had participated in a rural program at medical school were significantly more likely to provide a response at PGY3 than those who did not participate in a rural program (Table 4). Given the well-established positive association between rural immersion at medical school and the propensity of a graduate to practise outside of urban areas [31, 32], researchers using the PGY3 dataset in a longitudinal manner should be cognisant of this finding, in addition to the other ascribed variables for which significantly higher rates of drop-out were observed (Table 4), and account for these in their study design and analysis.

In conclusion, this study investigating the potential presence of non-response bias in the MSOD project has shown that samples collected during medical school (CMSQ, EQ) are representative and, therefore, findings that arise from using these datasets in a cross-sectional manner can be confidently generalised to the population of NZ medical graduates. The samples collected post-graduation (PGY1 and 3) are representative of the population on demographic variables, but not in the proportions of graduates represented from each university. It is important for researchers to be cognisant of this bias when interpreting their results when utilizing these data in particular. If the datasets are to be utilized in a longitudinal manner, such as comparing the change in career intentions or influences from one collection point to the next, then researchers may wish to employ analysis and modelling techniques that allow for them to control for ascribed variables and conduct a non-responders analysis. These findings would likely have general applicability to other similar studies in size and response rate [33, 34]. Future research could examine the representativeness of samples collected at later post-graduation time points. These were not examined in this study as only a very small proportion of the population of interest had been given the opportunity to respond to PGY5 and PGY8 surveys at the time of analysis. Additional case studies could be undertaken to explore the results of implementing missing data methods to adjust for the non-response bias detected in this data set. Overall, these findings have confirmed the value of the data collected as a part of the MSOD project and highlight its utility for answering questions relevant to current research fields in medical education and workforce development, provided appropriate considerations are made to account for non-response bias.

## Data Availability

The datasets supporting the conclusions of this article are available upon reasonable request from MSOD (MSOD.project@otago.ac.nz).

## List of Abbreviations

MSOD: Medical Schools Outcomes Database and Longitudinal Tracking Project
NZ: New Zealand
CMSQ: Commencing Medical Student Questionnaire
EQ: Exit Questionnaire
PGY1: Post-graduation Year One
PGY3: Post-graduation Year Three
PGY5: Post-graduation Year Five
PGY8: Post-graduation Year Eight
GP: General Practice
